# The Bu Shen Yi Sui Formula Promotes Axonal Regeneration *via* Regulating the Neurotrophic Factor BDNF/TrkB and the Downstream PI3K/Akt Signaling Pathway

**DOI:** 10.3389/fphar.2019.00796

**Published:** 2019-07-17

**Authors:** Qi Zheng, Lei Liu, Haolong Liu, Hong Zheng, Hao Sun, Jing Ji, Yaqin Sun, Tao Yang, Hui Zhao, Fang Qi, Kangning Li, Junling Li, Nan Zhang, Yongping Fan, Lei Wang

**Affiliations:** ^1^School of Traditional Chinese Medicine, Beijing Key Lab of TCM Collateral Disease Theory Research, Capital Medical University, Beijing, China; ^2^Department of Traditional Chinese Medicine, Beijing Tian Tan Hospital, Capital Medical University, Beijing, China; ^3^Oncology Department, Guang An Men Hospital of China Academy of Chinese Medical Sciences, Beijing, China; ^4^Physical Examination Department, The Chinese Medicine Hospital of Sanmenxia City, Henan, China

**Keywords:** Bu Shen Yi Sui formula, multiple sclerosis, model of nerve damage in SH-SY5Y cells, experimental autoimmune encephalomyelitis, axonal regeneration, BDNF/TrkB, PI3K/Akt pathways

## Abstract

Axonal damage is recognized as an important pathological feature in the chronic progressive neurological disorder multiple sclerosis (MS). Promoting axonal regeneration is a critical strategy for the treatment of MS. Our clinical and experimental studies have shown that the Bu Shen Yi Sui formula (BSYS) promotes axonal regeneration in MS and experimental autoimmune encephalomyelitis (EAE), an animal model of MS, but the exact mechanism has not been thoroughly elucidated to date. In this study, we investigated the effects of BSYS and its two decomposed formulas—the Bu Shen formula (BS) and the Hua Tan Huo Xue formula (HTHX)—on brain-derived neurotrophic factor (BDNF)/TrkB and related signaling pathways to explore the mechanism by which axonal regeneration is promoted *in vitro* and *in vivo*. Damaged SH-SY5Y cells incubated with low serum were treated with BSYS-, BS-, and HTHX-containing serum, and EAE mice induced by the myelin oligodendrocyte glycoprotein (MOG)_35–55_ peptide were treated with BSYS. The results showed that the BSYS-containing serum markedly increased cell viability and increased the levels of growth associated protein (GAP)-43, phosphorylated (p)-cAMP-response element binding protein (CREB), BDNF, TrkB, and p-PI3K. The BS and HTHX treatments also induced the protein expression of GAP-43 and p-extracellular signal-regulated kinase (ERK) in the cells. Furthermore, the effects of BSYS on cell viability, GAP-43, p-CREB, and neurite outgrowth were clearly inhibited by LY294002, a specific antagonist of the PI3K signaling pathways. The addition of U0126 and U73122, antagonists of the ERK and PLCγ pathway, respectively, significantly inhibited cell viability and GAP-43 protein expression. Moreover, BSYS treatment significantly increased the expression of the 68-, 160-, and 200-kDa neuroﬁlaments (NFs) of proteins and the BDNF, TrkB, PI3K, and Akt mRNA and proteins in the brain or spinal cord of mice at different stages. These results indicated that BSYS promotes nerve regeneration, and its mechanism is mainly related to the upregulation of the BDNF/TrkB and PI3K/Akt signaling pathways. BS and HTHX also promoted nerve regeneration, and this effect involved the ERK pathway.

## Introduction

Multiple sclerosis (MS) is an autoimmune response-mediated inflammatory neurodegenerative disease of the central nervous system (CNS) (Herz et al., 2010; Bowles et al., 2017). MS is characterized by demyelinating, axonal, and neuronal damage, which manifest as symptoms of muscle weakness, paraesthesia, numbness, and blurred vision in the clinical setting (Marrie et al., 2016; Peyro et al., 2016). Axonal injury plays a critical role in the severity of symptomatic progression in MS (Correale and Farez, 2015; Lee et al., 2015). Therefore, promoting axonal regeneration has been reported to be especially important in recent research (Alizadeh et al., 2015; Bove and Green, 2017).

Additional studies are underway to identify neurotrophic factors whose shortage results in difficulty repairing axons (Lingor et al., 2012; Razavi et al., 2015). Brain-derived neurotrophic factor (BDNF) is a small molecular peptide that plays an important role in the growth and development of neurons, the repair of damaged nerves, and the induction of axonal regeneration (Zhu et al., 2012; Wang et al., 2016). BDNF is closely associated with synapse formation and increases neurotransmitter release from the synaptic cleft, subsequently stimulating the growth of axons (Brady et al., 2018; Niu et al., 2018). In addition, BDNF activates three main signal transduction pathways, PI3K, extracellular signal-regulated kinases (ERK), and PLCγ, after binding to its receptor TrkB in presynaptic terminals (Zhu et al., 2012). Furthermore, BDNF has neuroprotective effects through regulating the downstream transcription factor cAMP-response element binding protein (CREB) (De Santi et al., 2009). Clinical observation found low levels of BDNF expression in MS patients (Kalinowska-Łyszczarz et al., 2018). Experimental studies also found decreased expression of BDNF in the brain and spinal cord in mice with experimental autoimmune encephalomyelitis (EAE), an animal model of MS (Wang et al., 2014). Exogenous BDNF prolonged latency and improved neurological function scores in EAE mice (Makar et al., 2008). Activation of the PI3K/Akt signaling pathway helped to stabilize the internal milieu in the brain and protect neurons, which could accelerate angiogenesis and resistance to oxidative stress in MS patients. LY294002 inhibited the PI3K subunit p110, which prevented the activation of PI3K. Therefore, LY294002 was recognized as an inhibitor of the PI3K/Akt signaling pathway. This study showed that ERK expression was rapidly activated after nerve damage to reduce apoptosis and injury. ERK was markedly decreased after U0126 treatment (Zhang et al., 2015b), and activation of PLCγ was significantly decreased by U73122 treatment (Liu et al., 2015), which inhibited the pathway. Thus, regulation of neurotrophic factors and their related signaling pathways to promote axonal regeneration is the key to resolving CNS damage in MS.

The current therapeutic agents for MS include corticosteroids and immunosuppressants, which control flares of MS (Di et al., 2018; Kim et al., 2018). However, these therapies are ineffective in controlling relapse rates and have potential side effects (Compston and Coles, 2008; Harlow et al., 2015). There is no effective neuroprotective therapy for MS; as such, multifaceted treatment strategies are required (Pifarre et al., 2014; Gholamzad et al., 2019). Traditional Chinese medicine (TCM) has been shown to be effective in resolving clinical symptoms, reducing recidivism, regulating immune function, and promoting remyelination and axonal regeneration in the treatment of MS (Pan et al., 2013; Salmen and Chan, 2015). The Bu Shen Yi Sui formula (BSYS) is a Chinese herbal formula created by YF based on Liuwei Dihuang Pills (a well-known formula in the Song dynasty). BSYS consists of two parts, the medicine Bu Shen (BS, which refers to tonifying the kidney) and the medicine Hua Tan Huo Xue (HTHX, refers to dissolving phlegm and activating blood circulation), and it has been effectively and exclusively applied for the treatment of MS for 15 years in China (Fang et al., 2013a; Zhou and Fan, 2015). Previous clinical studies showed that BSYS has the ability to markedly reduce and eliminate symptoms such as limb weakness and paraesthesia, reduce the frequency and intensity of relapses, ameliorate the side-effects of prednisone (PA), and reduce the dose of medication required, to improve quality of life in MS patients. BSYS has been approved by the Beijing Food and Drug Administration as a hospital preparation (No. 10003). Several *in vivo* pharmacological studies have found that BSYS have neuroprotective effects (Fang et al., 2013b; Li et al., 2013; Zheng et al., 2015), and *in vitro* studies have also shown that BSYS can promote axonal outgrowth in SH-SY5Y cells (Liu et al., 2014). However, the mechanism by which BSYS promotes axonal regeneration is unclear.

In this study, the effects of BSYS and its decomposed BS and HTHX formulas on BDNF/TrkB and related signaling pathways were investigated in SH-SY5Y cells and EAE mice. Cell viability was measured with the cell counting kit (CCK)-8 assay, neurite outgrowth was observed by immunofluorescence (IF), and the protein expression of growth-associated protein (GAP)-43, CREB, BDNF, TrkB, PI3K, ERK, and PLCγ was observed by Western blotting (WB) *in vitro*. Expression levels of neurofilament protein (NF) 200 were measured by IF, and the mRNA and protein expression levels of BDNF, TrkB, PI3K, and Akt in the brain and spinal cord of mice were detected by real-time quantitative reverse transcription polymerase chain reaction (qRT-PCR) and WB *in vivo*.

## Materials and Methods

### Chemicals and Medicines

DMEM-F12, fetal bovine serum, trypsin, *Mycobacterium tuberculosis* strain H37Ra (MTB), LY294002, U0126, and U73122 were purchased from Gibco BRL (Grand Island, NY, USA). The myelin oligodendrocyte glycoprotein (MOG)_35–55_ peptide (MEVGWYRSPFSRVVHLYRNGK; purity >95%) was synthesized by Beijing Xuheyuan Biotech Co., Ltd. (Beijing, China). Complete Freund’s adjuvant (CFA) and pertussis toxin (PTX) were purchased from Sigma-Aldrich (St. Louis, MO, USA). Antibodies specific for β-tubulin-III, NF200, GAP-43, phosphorylated (p)-CREB, ERK, p-ERK, PLCγ, TrkB, PI3K, p-PI3K, Akt, and p-Akt were purchased from Cell Signaling Technology (Danvers, MA, USA); antibodies specific for CREB, NF68, and NF160 were purchased from Abcam (Cambridge, MA, UK), and the antibody specific for BDNF antibody was purchased from Epitomics (Burlingame, USA). The sheep anti-rabbit-FITC (fluorescein isothiocyanate) secondary antibody and DAPI were purchased from Beijing Biosynthesis Biotechnology Co. Ltd. (Beijing, China). qRT-PCR kits and reverse transcription kits were purchased from Tiangen Biotech Co., Ltd. (Beijing, China). PCR primers were synthesized by TaKaRa Biotechnology Co. Ltd. (Dalian, China). WB kits were purchased from Applygen Technologies Inc. (Beijing, China). The herbal medicines for BSYS were purchased from Beijing Ya Dong Biological Pharmacy Co., Ltd. (Beijing, China). Prednisone (PA) was purchased from Zhejiang Xianju Pharmaceutical Co., Ltd. (Zhejiang, China). Forsythoside E, 2,3,5,4′-tetrahydroxyl diphenylethylene-2-O-glucoside, and forsythin were purchased from the National Institutes for Food and Drug Control (Beijing, China).

### BSYS Preparation

BSYS was composed of *Rehmanniae Radix* (Rehmannia root), *Rehmanniae Radix* Praeparata (processed Rehmannia root), *Polygoni Multiflori Radix* (fleeceflower root), *Rhei Radix et Rhizoma* (rhubarb), *Leonuri Herba* (motherwort herb), *Fritillariae Thunbergii Bulbus* (Thunberg fritillary bulb), *Hirudo* (leech), *Scorpio* (scorpion), *Gastrodiae Rhizoma* (tall Gastrodia tuber), and *Forsythiae Fructus* (weeping forsythia capsule). The ratio of these herbs was 10:10:10:2:10:6:3:2:3:6. BSYS was decomposed into two parts, namely, the BS formula including *Rehmanniae Radix* (Rehmannia root), Rehmanniae Radix Praeparata (processed Rehmannia root), and *Polygoni Multiflori Radix* (Fleeceflower root), and the HTHX formula including *Rhei Radix et Rhizoma* (rhubarb), *Leonuri Herba* (motherwort herb), *Fritillariae Thunbergii Bulbus* (Thunberg fritillary bulb), *Hirudo* (leech), *Scorpio* (scorpion), *Gastrodiae Rhizoma* (tall Gastrodia tuber), and *Forsythiae Fructus* (weeping forsythia capsule). The main compounds and the chemical characteristic fingerprinting of BSYS were identified with ultra-performance liquid chromatography–quadrupole time-of-flight mass spectrometry (UPLC-QTOF-MS). The detailed methods for preparation and quality control of BSYS have been previously published (Zheng et al., 2015). In this study, the same batch of BSYS was used. The proportions of the herbal medicines in BS and HTHX and the preparation process were the same as those described for BSYS.

### Cells and Animals

SH-SY5Y cells were provided by Prof. Xiaomin Wang, Key Laboratory for Neurodegenerative Disorders of the Ministry of Education. Specific pathogen-free (SPF)-grade male Sprague–Dawley (SD) rats (*n* = 60) weighing 180–220 g and female C57BL/6 mice (*n* = 80) weighing 18–22 g were obtained from the Beijing Vital River Laboratories, Beijing, China. The mice were fed in the Center of Laboratory Animals at Capital Medical University. The mice were housed under a 12-h light/dark cycle in individual ventilated cages and maintained in an SPF-grade environment. The studies were conducted in accordance with the Guide for Laboratory Animal Care by the Ethics Committee of Capital Medical University.

### Ethics Statement

All animal studies and procedures were approved by the Ethics Committee of Capital Medical University. The animals were anesthetized by intraperitoneal injection of a solution containing 10% chloral hydrate (350 mg/kg body weight).

### Preparation of BSYS-, BS-, and HTHX-Containing Serum

After an acclimation period of three days, the rats were randomly divided into four groups: the blank control serum (BCS, serum without Chinese herbal medicines), BSYS-containing serum, BS-containing serum, and HTHX-containing serum groups. There were 15 rats in each group. The rats in the BSYS, BS, and HTHX groups received intragastric administration of the indicated treatments at doses of 11.7 g/kg, 6.45 g/kg, and 4.45 g/kg twice a day for 7 days. The rats in the BCS group were given the same amount of normal saline (NS) with the same procedure. All rats were anesthetized with 4% chloral hydrate after the last administration, and blood was collected through the left ventricle of rats. The serum was isolated by centrifugation at 3,000 rpm for 20 min after incubation at 4°C for 2 h, and the serum was inactivated at 56°C for 30 min and filtered with a 0.22-µm pore-size membrane. The serum was kept frozen at −80°C until use. To ensure the quality and stability of BSYS-, BS-, and HTHX-containing serum, ultra-performance liquid chromatography–mass spectrometry (UPLC-MS/MS) was used to identify the active ingredients.

### Determination of the Main Chemical Constituents in BSYS-, BS-, and HTHX-Containing Serum by UPLC-MS/MS

Identification analysis was performed on a DIONEX UltiMate 3000 UPLC system (Thermo Fisher Scientific, San Jose, CA, USA) coupled to an LTQ-Orbitrap mass spectrometer *via* an ESI interface. Samples were separated on an Agilent poroshell 120 EC-C18 reverse-phase column (4.6 mm × 150 mm, 2.7 μm). Mobile phase A was water (containing 0.1% formic acid), and mobile phase B was acetonitrile. The gradient elution program was as follows: 0→5 min, 5% B; 5→10 min, 5→30% B; 10→25 min, 30→60% B; 25→40 min, 60→80% B; 40→45 min, 80→95% B. The flow rate was 0.3 ml/min, and the column temperature was 25°C. The injection volume was 10 μl.

The mass spectrometer was operated in negative mode. The spray voltage was 5.0 kV, and the S-lens was set at 60%. The ESI source temperature and capillary temperature were 300°C and 350°C, respectively. The sheath gas and auxiliary gas were high-purity nitrogen, and the flow rates were 50 arb and 5 arb, respectively. The scan range was from 110 to 2,000 Da, and the resolution was 30,000.

**Figure 1:**
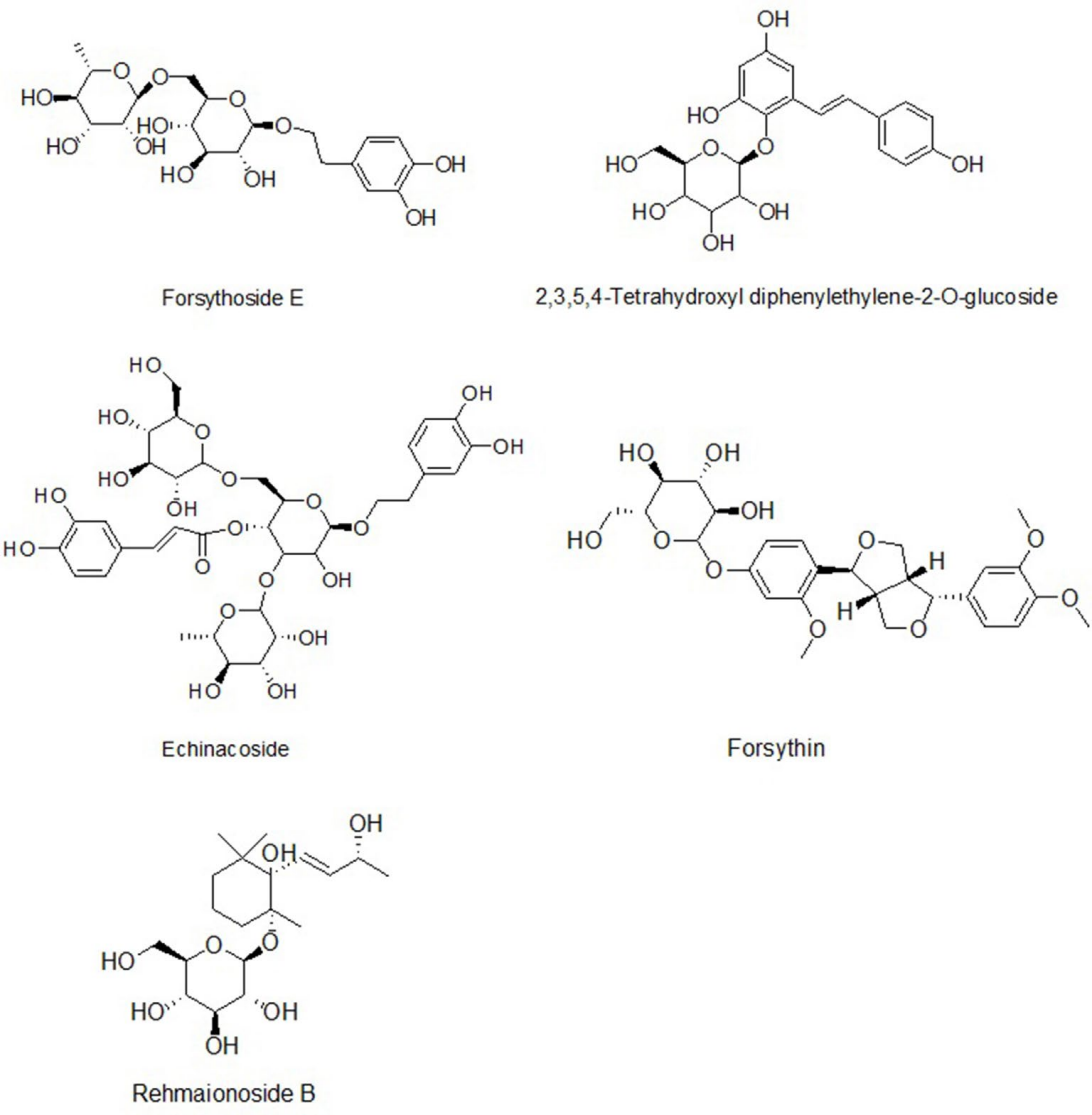
The structures of five compounds in rat serum.

**Figure 2:**
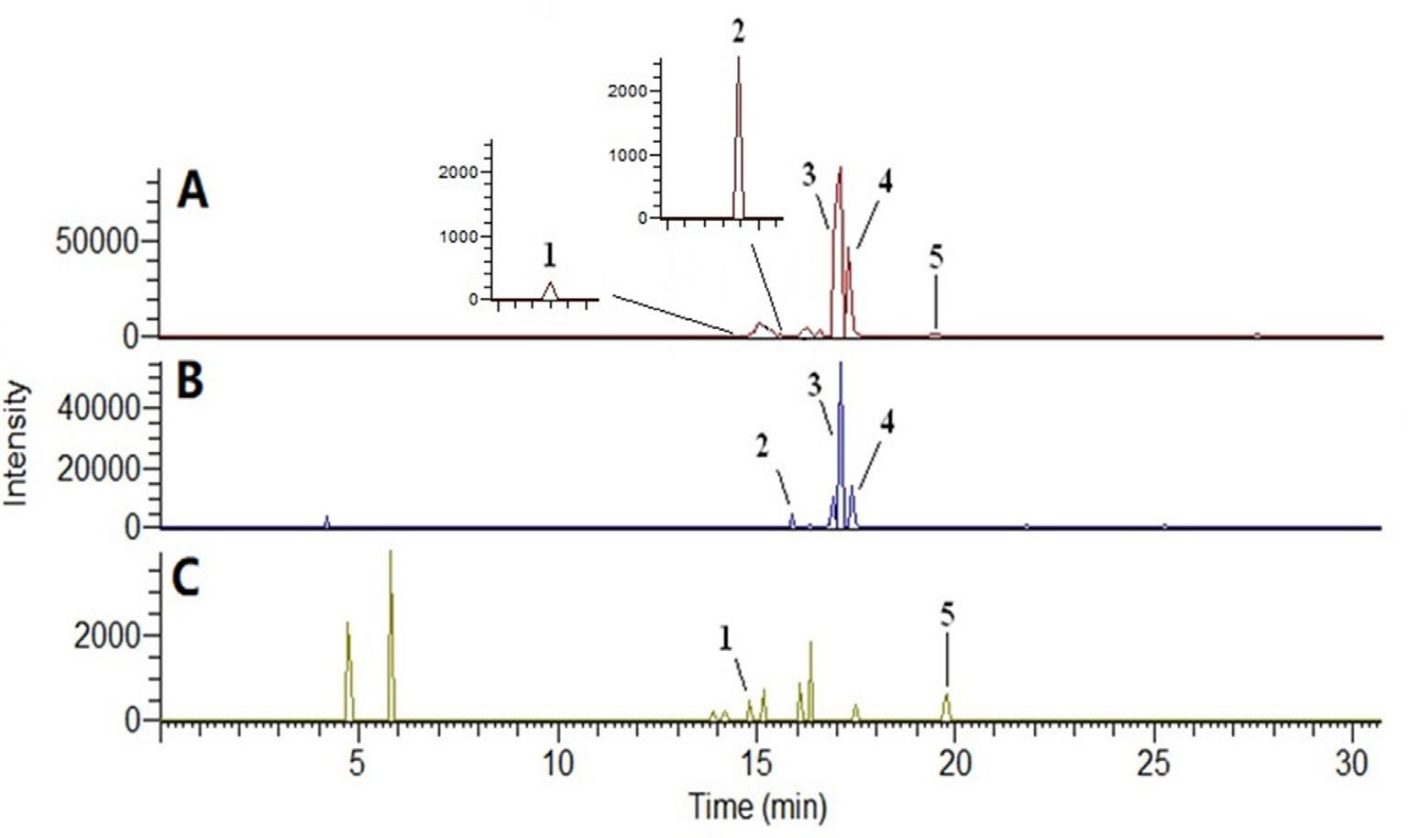
Base peak ion chromatogram of plasma samples in negative ion mode by UPLC-MS/MS. **(A)** Bu Shen Yi Sui formula (BSYS) group; **(B)** Bu Shen formula (BS) group; **(C)** Hua Tan Huo Xue formula (HTHX) group. (1) Forsythoside E; (2) echinacoside; (3) rehmaionoside B; (4) 2,3,5,4′-tetrahydroxyl diphenylethylene-2-O-glucoside; (5) forsythin.

The compounds identified were forsythoside E, echinacoside, rehmaionoside B, 2,3,5,4′-tetrahydroxyl diphenylethylene-2-O-glucoside, and forsythin, and they are shown in **Figures 1** and **2** and **Table 1**.

**Table 1:** Data for five components identified by ultra-performance liquid chromatography–mass spectrometry (UPLC-MS/MS).

No.	*t*_R_	Precursor ion (*m/z*)	Elem. comp.	Diff. (ppm)	Identification
1	14.29	[M-H]^-^	C_20_H_29_O_12_	−5.709	Forsythoside E^a^
		461.1627			
2	15.74	[M-H+CH_3_COOH]^-^	C_36_H_47_O_20_	−5.067	Echinacoside^a^
		799.2615			
3	16.90	[M-H+CH_3_COOH]^-^	C_21_H_37_O_10_	−5.150	Rehmaionoside B
		449.2358			
4	17.29	[M-H]^-^	C_20_H_21_O_9_	−6.094	2,3,5,4′-Tetrahydroxyl diphenylethylene-2-O-glucoside^a^
		405.1155			
5	19.83	[M-H+CH_3_COOH]^-^	C_29_H_37_O_13_	−3.957	Forsythin^a^
		593.2205			

a Structures were confirmed by comparing retention time and high-resolution accurate mass values to reference standards.

### Quantification of Forsythoside E, 2,3,5,4′-Tetrahydroxyl diphenylethylene-2-O-glucoside, and Forsythin in the BSYS, BS, and HX Formulas

Aliquots of 500 μl of rat serum were mixed with 1000 μl of acetonitrile. The samples were vortexed for 1 min and then centrifuged at 12,000 *g* for 10 min at 4°C. Aliquots of 10 μl of the supernatant were used in the LC-MS/MS analysis.

The concentrations of forsythoside E (CID: 69634125), 2,3,5,4′-tetrahydroxyl diphenylethylene-2-O-glucoside (CID: 5321884), and forsythin (CID: 101712) in the BSYS formula were 6.02 μg/g, 32.20 μg/g, and 8.69 μg/g, respectively. Forsythoside E and forsythin were undetected in the BS formula, while the concentration of 2,3,5,4′-tetrahydroxyl diphenylethylene-2-O-glucoside in the BS formula was 22.57 μg/g. Forsythoside E and 2,3,5,4′-tetrahydroxyl diphenylethylene-2-O-glucoside were undetected in the HX formula, while the concentration of forsythin in the HT formula was 5.52 μg/g.

### Cell Culture and Treatment

SH-SY5Y cells were cultured in DMEM-F12 supplemented with 10% FBS, 100 U/ml penicillin, and 100 mg/ml streptomycin at 37°C in a 5% CO_2_ atmosphere. The cells were maintained in low serum medium [0.25% fetal bovine serum (FBS)] for 24 h to establish a model of nerve damage according to the corresponding references (Lai et al., 2011; Klinkenberg et al., 2012). A previous study found that 20% BSYS-containing serum significantly promoted axonal outgrowth in SH-SY5Y cells (Liu et al., 2014). Therefore, 20% BSYS-containing serum was used in subsequent experiments.

To observe the effects of BSYS-containing serum on cell viability with different incubation times, the cells were seeded in 96-well plates at a density of 3 × 10^4^ cells per well in 100 μl of medium. The cells were divided into four groups: normal control (NC), model (MO), MO + BCS (BCS), and MO + BSYS (BSYS). The cells in the NC group were cultured in DMEM-F12 supplemented with 10% FBS, and the cells in the MO, BCS, and BSYS groups were cultured in DMEM-F12 with 0.25% FBS. The cells in the BCS and BSYS groups were treated with 20% BCS- and BSYS-containing serum, respectively, and after 12 h, 24 h, and 48 h, the cell viability was determined with the CCK-8 assay. To observe the effects of BSYS-containing serum and the inhibitors LY294002, U0126, and U73122, the cells were divided into six groups: NC, MO, MO + BSYS (BSYS), MO + LY294002 + BSYS (LY294002), MO + U0126 + BSYS (U0126), and MO + U73122 + BSYS (U73122). The cells in the last three groups were pre-treated for 1 h with the inhibitors LY294002 (60 μmol), U0126 (10 μmol), and U73122 (0.5 μmol) or vehicle (0.1% DMSO), followed by exposure to BSYS-containing serum for 24 h. The cell viability was measured with the CCK-8 assay, and neurite outgrowth was measured with an immunofluorescence (IF) assay.

To observe the effects of BSYS-, BS-, and HTHX-containing serum on axonal regeneration, the cells were seeded in 100-mm dishes at a density of 5 × 10^7^ cells per dish in 8 ml of medium. The cells were divided into five groups: NC, MO, MO + BSYS (BSYS), MO + BS (BS), and MO + HTHX (HTHX). The cells in all groups were washed with PBS, scraped in ice-cold protease inhibitor cocktail with PMSF, and incubated on ice for 1 h. The cellular debris was removed by centrifugation (15,000 rpm for 20 min) at 4°C. The protein concentration was measured with the BCA method, and the samples were then analyzed with WB.

### EAE Model Establishment and Treatment

The mice were randomly divided into four groups: NC (*n* = 20), EAE model (EAE, *n* = 20), EAE + PA-treated (PA, *n* = 20), and EAE + BSYS-treated (BSYS, *n* = 20). The EAE mice were injected subcutaneously (s.c.) with 0.2 ml of an emulsion containing 50 μg MOG_35–55_ in 100 μl of CFA and 100 μl of NS, followed by intraperitoneal (i.p.) injections of 500 ng of PTX on Days 0 and 2 post-induction (PI) (Liu et al., 2012). In this study, the mice in the BSYS group were given an oral suspension of 3.02 g/kg BSYS, which was effective for the treatment of EAE in our previous study (Fang et al., 2013b; Zheng et al., 2015) when administered once per day for 40 days. The mice in the PA group were administered PA at a dose of 6 mg/kg. The NC and EAE mice were treated with NS. The mice were sacrificed on Days 18 (acute stage) and 40 (remission stage) PI. The brain and spinal cord were immediately frozen for qRT-PCR and Western blot analysis, and 4% paraformaldehyde was used to fix the brain and spinal cord for IF analysis.

### Animal Behavior Test

The neurological function scores were applied as a method of animal behavior test in the experiment. The scores were observed once a day based on the sum of the disease state for the tail and all four limbs as follows: 0, no signs of disease in the tail and limbs; scores for the tail were assigned as 1 for paralyzed tail tip paralysis and 2 for tail flaccidity; scores for the limbs were assigned as 1 for gait disturbance, 2 for moderate paralysis and limb dragging, and 3 for total paralysis. Mortality was assigned a score of 15 (Dasilva and Yong, 2008).

### Cell Viability Assay

Cell viability was measured with the CCK-8 assay. After the cells were treated with different methods, 10 μl of CCK-8 reagent was added to the cells, and the cells were cultured for an additional 4 h. The optical density (OD) was measured at 450 nm. Cell viability = OD value of all groups/OD value of NC group ×100%.

### IF Staining and Analysis

The cells were seeded in 24-well plates at a density of 5 × 10^5^ cells per well in 500 μl of medium. After the cells were treated with different methods, cells at 80% confluence on cover slips were fixed with 4% paraformaldehyde. Samples of brain and spinal cord were prepared after fixation in 4% paraformaldehyde and embedding in paraffin. The slides were incubated with primary detection antibodies [rabbit anti-human β-tubulin-III (1:600) and rabbit anti-mouse NF200 (1:200)] at 4°C for 16 h. Subsequently, the slices were washed three times with PBS and incubated with the secondary antibody (sheep anti-rabbit-FITC, 1:800) at 37°C for 60 min and then washed three times with PBS. The slices were counter-stained with DAPI and maintained at 4°C. Finally, the slices were dehydrated and mounted for microscopic observation. Ten high-power fields (400×) were selected from five slices. For cells, the percentage of the total number of cells (at least 100 cells) bearing neurites was calculated. Neurite length was measured by the NIS-Elements BR 3.2 software. Quantitative analysis of the IF-stained images was carried out using a Leica TCS-SP5 Confocal Microscope (Leica Microsystems, Wetzlar, Germany), and the results were expressed as integral optical density (IOD) values.

### Western Blot Analysis

Protein extraction and quantification were performed according to the procedures specified by the manufacturers of the reagents used. Each sample containing 20 μg of protein was separated by 5% and 10% SDS-PAGE and electrotransferred onto polyvinylidene fluoride membranes (Millipore, USA). The membranes were incubated with a primary rabbit anti-GAP-43 antibody (1:10,000), rabbit anti-CREB antibody (1:1,000), rabbit anti-p-CREB antibody (1:1,000), rabbit anti-BDNF antibody (1:20,000), rabbit anti-TrkB antibody (1:10,000), rabbit anti-PI3K antibody (1:1,000), rabbit anti-p-PI3K antibody (1:1,000), rabbit anti-Akt antibody (1:1,000), rabbit anti-p-Akt antibody (1:1,000), rabbit anti-ERK antibody (1:1,000), anti-p-ERK antibody (1:1,000), rabbit anti-PLCγ antibody (1:10,000), rabbit anti-NF200 antibody (1:1,000), rabbit anti-NF68 antibody (1:20,000), rabbit anti-NF160 antibody (1:1,000), rabbit polyclonal anti-β-tubulin antibody (1:50,000), or rabbit polyclonal anti-GAPDH antibody (1:20,000) in blocking solution at 4°C overnight. Then, the membranes were incubated with secondary goat anti-rabbit IgG (1:20,000) for 60 min and electrochemiluminescence (ECL) reagent for 30 s to 2 min followed by exposure to Kodak film (Japan). Data were represented by the IOD ratio determined using the ImageQuant TL 2005 image analysis software (Amersham, Biosciences, Piscataway, NJ).

### qRT-PCR Analysis

Total RNA was isolated from approximately 30 mg of brain or spinal cord tissue from mice according to the manufacturer’s instructions. RNA samples with an OD_260_/OD_280_ ratio of 1.9–2.1 and an OD_260_/OD_230_ ratio greater than 2.0 were used for analysis. cDNA was synthesized from total RNA by reverse transcription of 1 μg of total RNA using a reverse transcription kit. The primer sequences for PCR were designed by the Primer Premier 5.0 software based on the GenBank sequences and are shown in **Table 2**. PCRs were carried out using the following conditions: 95°C for 15 min, followed by 40 cycles of denaturation at 95°C for 10 s, annealing at 52°C for 30 s (BDNF, TrkB) or 56°C for 30 s (PI3K, Akt), and extension at 72°C for 31 s (Applied Biosystems 7300, Foster, USA). Relative quantification was performed by the 2^−ΔΔCt^ method.

**Table 2:** Primer sequences of mRNAs for reverse transcription polymerase chain reaction (RT-PCR).

Target gene	Primer	Length (bp)
BDNF (NM_001270630.1)	F, 5′-CAGCGCGAATGTGTTAGTGGTTA-3′	112
	R, 5′-CAGTGGACAGCCACTTTGTTTCA-3′	
TrkB (NM_001163168.2)	F, 5′-GTGGATTCCGGCTTAAAGTTTGTG-3′	126
	R, 5′-AAGTCAAGGTGGCGGAAATG-3′	
PI3K (NM_001024955.2)	F, 5′-CCCATGGGACAACATTCCAA-3′	139
	R, 5′-CATGGCGACAAGCTCGGTA-3′	
Akt (NM_001165894.1)	F, 5′-TCAGGATGTGGATCAGCGAGA-3′	112
	R, 5′-CTGCAGGCAGCGGATGATAA-3′	
β-actin (NM_007393.3)	F, 5′-CTGAAAATCAATAGCACGAAC-3′	171
	R, 5′-ATGGAGCCACCGATCCACA-3′	

### Statistical Analysis

The results were expressed as the mean ± SE. Differences between groups were compared with one-way ANOVA with SPSS version 17.0 (SPSS Inc., Chicago, IL, USA). *P* values < 0.05 were considered statistically significant.

## Results

### Effects of BSYS-, BS-, and HTHX-Containing Serum on Cell Viability in SH-SY5Y Cells

**Figure 3:**
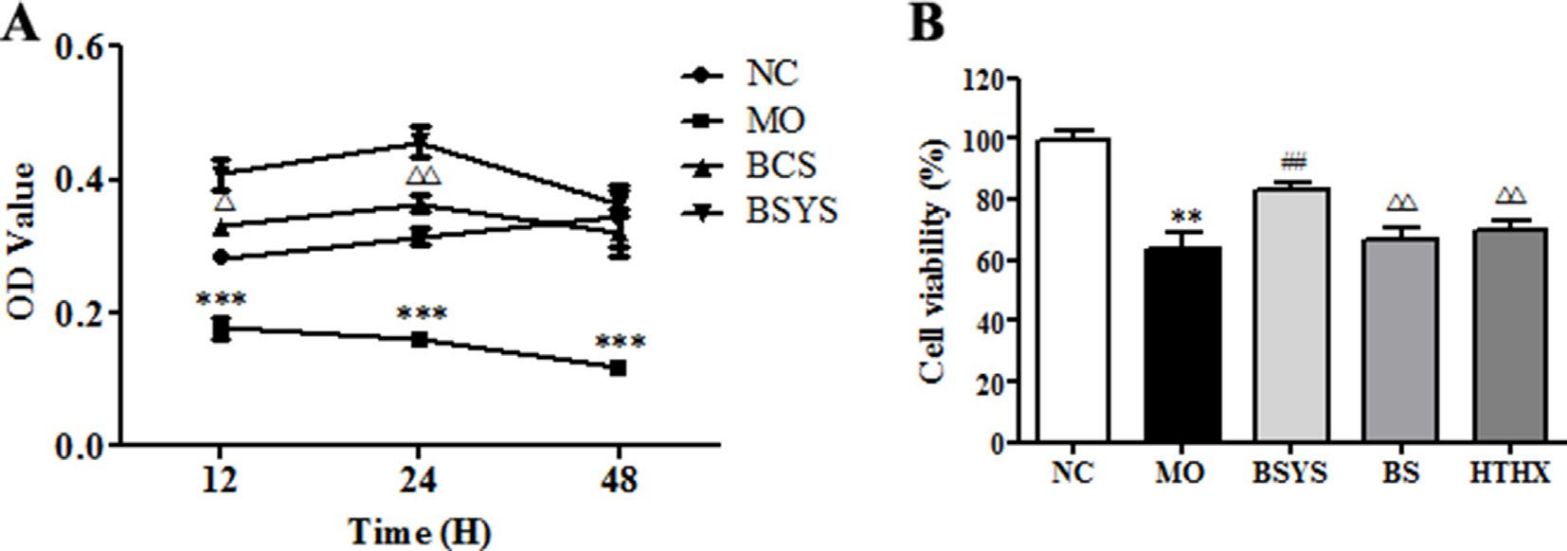
Effects of BSYS-, BS-, and HTHX-containing serum on the cell viability in SH-SY5Y cells. SH-SY5Y cells (3 × 10^4^/well) were seeded in 96-well plates in normal medium [10% fetal bovine serum (FBS)] or low-serum medium (0.25% FBS) for 24 h prior to exposure to blank control serum (BCS) or BSYS-containing serum for an additional 12 h, 24 h, or 48 h **(A)**, and BSYS-, BS-, and HTHX-containing serum for an additional 24 h **(B)**. The cell viability was determined by a CCK-8 assay, as described in the Materials and Methods, and expressed as the optical density (OD) value. Data represent the mean ± SE. *n* = 6. **P < 0.01, ***P < 0.001 vs. NC; ^##^ *P* < 0.01 vs. MO; ^△^ *P* < 0.05, ^△△^ *P* < 0.01 vs. BSYS.

Relative cell viability was analyzed using the CCK-8 assay. As shown in **Figure 3A**, cell viability in the NC group gradually increased as the incubation time with serum was extended (12, 24, or 48 h). Cell viability in the MO group decreased significantly compared to that in the NC group (*P* < 0.001). After the cells were treated with BCS- or BSYS-containing serum, the cell viability values reached a peak at 24 h. Cell viability in the BSYS group was higher than that in the BS group at the time points of 12 and 24 h (*P* < 0.05, *P* < 0.01), especially at 24 h. Therefore, 24 h of incubation with BSYS, BS, and HTHX was used in subsequent experiments (*P* < 0.01). **Figure 3B** showed that the cell viability in the MO group was significantly lower than in the NC group (*P* < 0.01), while the cell viability was significantly increased in the BSYS group compared to that in the MO, BS, and HTHX groups (*P* < 0.01).

### Effects of BSYS-, BS-, and HTHX-Containing Serum on GAP-43 and CREB Protein Expression in SH-SY5Y Cells

**Figure 4:**
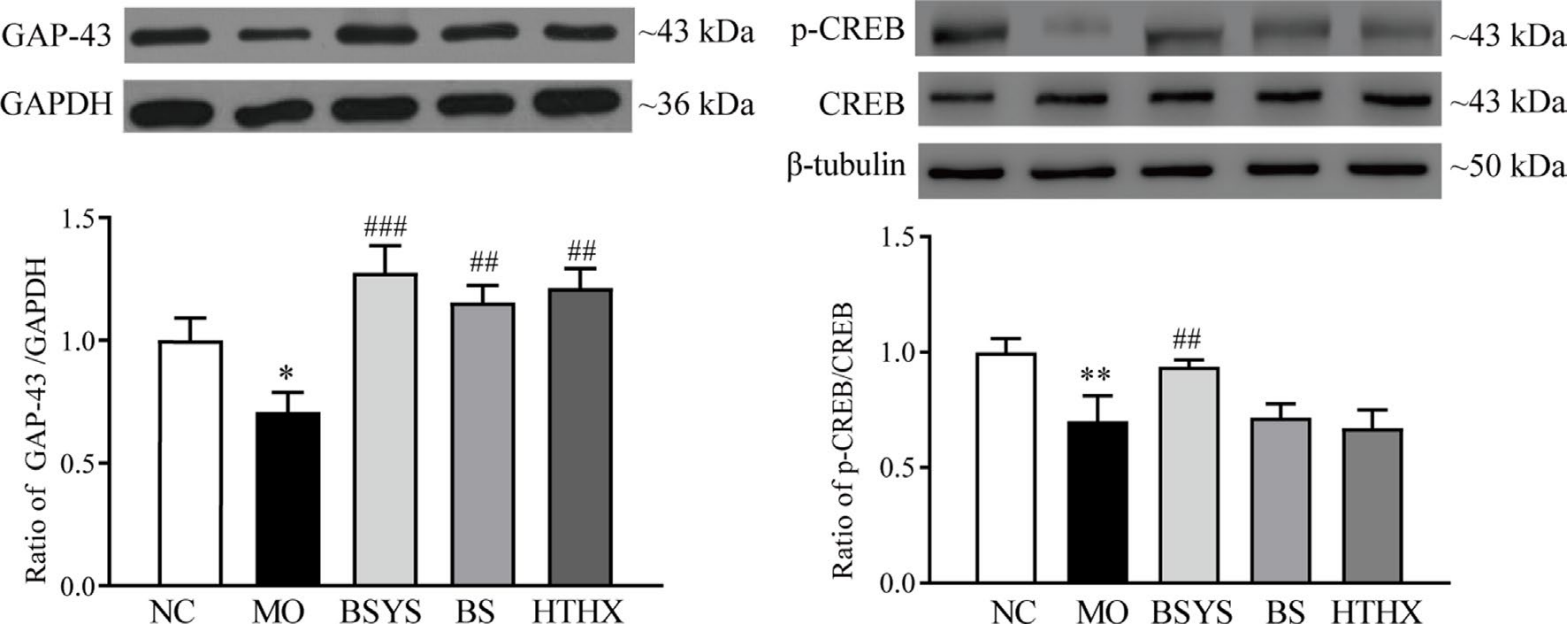
Effects of BSYS-, BS-, and HTHX-containing serum on GAP-43 and cAMP-response element binding protein (CREB) protein expression in SH-SY5Y cells. SH-SY5Y cells (5 × 10^7^/dish) were seeded in 100-mm dishes in normal medium (10% FBS) for 48 h and then shifted to low-serum medium (0.25% FBS) prior to exposure to BCS- or BSYS-containing serum for an additional 24 h. GAP-43 and p-CREB/CREB protein expression was detected by Western blotting (WB), as described in the Materials and Methods. Data represent the mean ± SE, *n* = 4. *P < 0.05, **P < 0.01 vs. NC; ^##^ *P* < 0.01, ^###^ *P* < 0.001 vs. MO; ^△△^ *P* < 0.01 vs. BSYS.

GAP-43, p-CREB, and CREB protein expression was determined by Western blot analysis. **Figure 4** shows that GAP-43 protein and the p-CREB/CREB ratio in the MO group were significantly downregulated compared to those in the NC group (*P* < 0.05, *P* < 0.01). GAP-43 levels and the p-CREB/CREB ratio in the BSYS group are significantly upregulated compared to those in the MO group (*P* < 0.001, *P* < 0.01). Only the BS and HTHX treatments increased GAP-43 protein expression (*P* < 0.01). The effect of BSYS on increase of CREB was better than that of BS and HTHX (*P* < 0.01).

### Effects of BSYS-, BS-, and HTHX-Containing Serum on BDNF and TrkB Protein Expression in SH-SY5Y Cells

**Figure 5:**
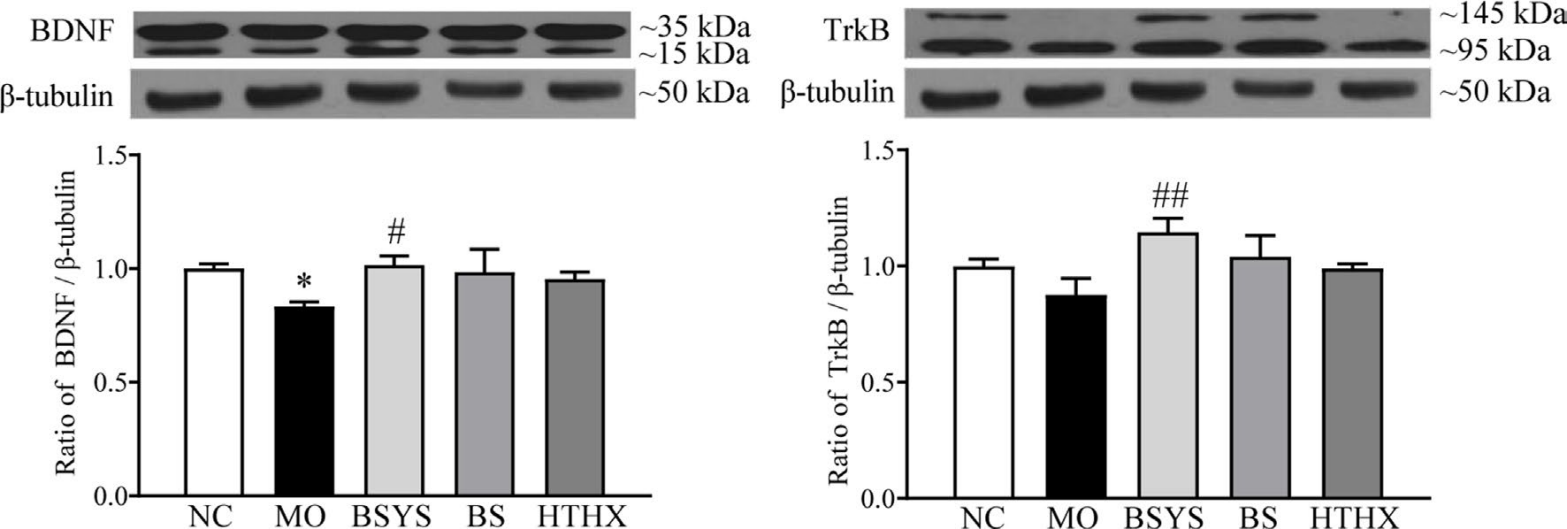
Effects of BSYS-, BS-, and HTHX-containing serum on brain-derived neurotrophic factor (BDNF) and TrkB protein expression in SH-SY5Y cells. SH-SY5Y cells (5 × 10^7^/dish) were seeded in 100-mm dishes in normal medium (10% FBS) for 48 h and then shifted to low-serum medium (0.25% FBS) prior to exposure to medicine-free serum or serum containing medicines from BSYS for an additional 24 h. BDNF and TrkB protein expression was detected by WB, as described in the Materials and Methods. Data represent the means ± SE, *n* = 4. *P < 0.05 vs. NC; ^#^ *P* < 0.05, ^##^ *P* < 0.01 vs. MO.

As shown in **Figure 5**, BDNF protein expression in the MO group was significantly downregulated compared to that in the NC group (*P* < 0.05); TrkB protein expression was not significantly different between the two groups. In comparison, the BDNF and TrkB protein levels in the BSYS group were significantly upregulated compared to those in the MO group (*P* < 0.05, *P* < 0.01). There were no significant changes in BDNF or TrkB levels in the BS and HTHX groups.

### Effects of BSYS-, BS-, and HTHX-Containing Serum on PI3K, ERK, and PLCγ Protein Expression in SH-SY5Y Cells

**Figure 6:**
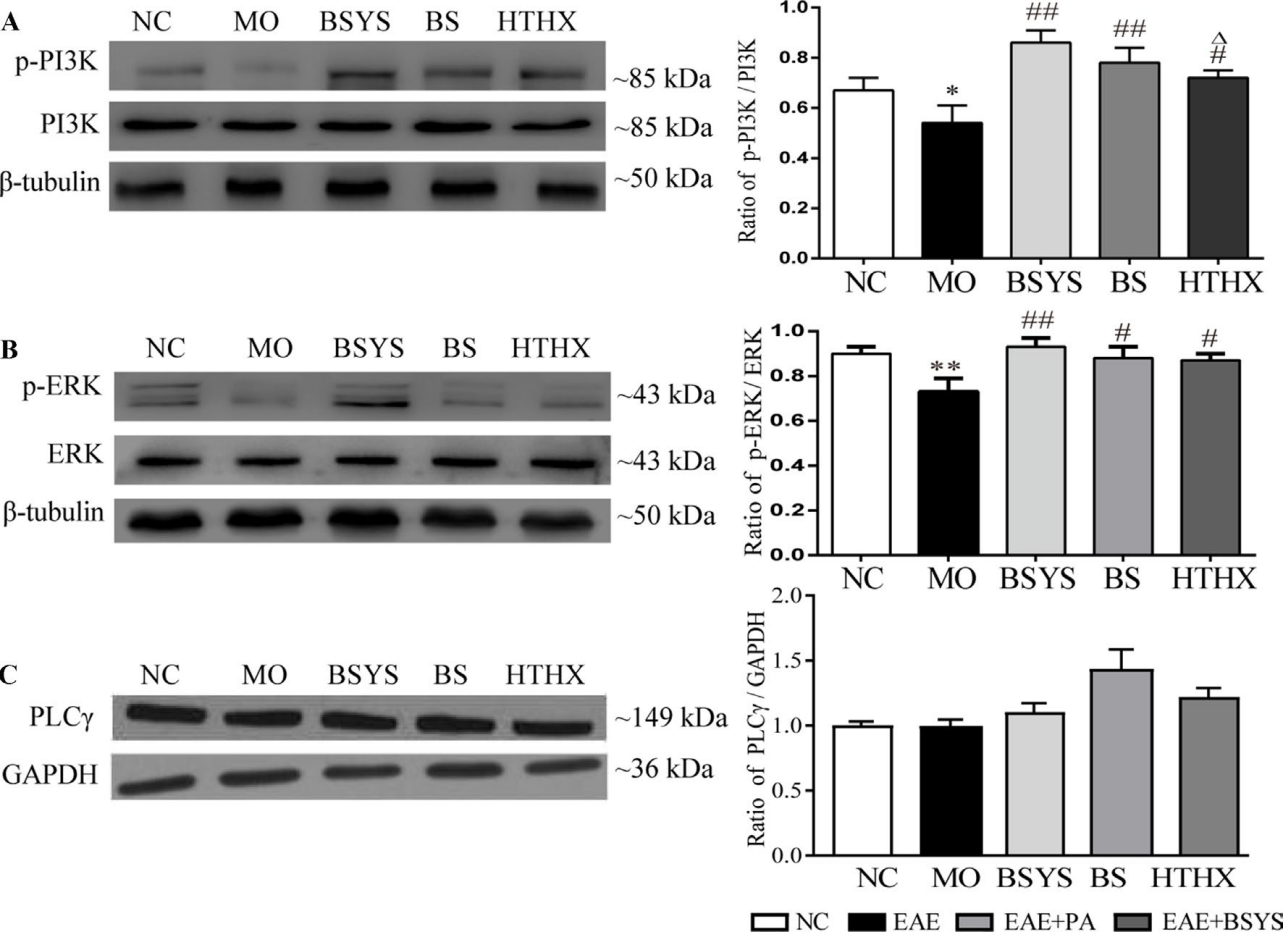
Effects of BSYS-, BS-, and HTHX-containing serum on PI3K, ERK, and PLCγ protein expression in SH-SY5Y cells. SH-SY5Y cells (5 × 10^7^/dish) were seeded in 100-mm dishes in normal medium (10% FBS) for 48 h and then shifted to low-serum medium (0.25% FBS) for 24 h prior to exposure to medicine-free serum or serum containing medicines from BSYS for an additional 24 h. P-PI3K/PI3K **(A)**, p-ERK/ERK **(B)** and PLCγ **(C)** protein expression was detected by WB as described in the Materials and Methods. Data represent the mean ± SE, *n* = 4. *P < 0.05, **P < 0.01 vs. NC; ^#^ *P* < 0.05, ^##^ *P* < 0.01 vs. MO; ^△^ *P* < 0.05 vs. BSYS.

As shown in **Figure 6**, the ratio of p-PI3K/PI3K and p-ERK/ERK in the MO group was significantly reduced compared to that in the NC group (*P* < 0.05, *P* < 0.01). The ratios of p-PI3K/PI3K and p-ERK/ERK were increased in the BSYS, BS, and HTHX groups compared to that in the MO group (*P* < 0.05, *P* < 0.01). In addition, BSYS treatment increased p-PI3K/PI3K ratio significantly more than HTHX treatment (*P* < 0.05). There was no significant difference in PLCγ levels among the NC, MO, BSYS, BS, and HTHX groups.

### Effects of BSYS-Containing Serum and the LY294002, U0126, and U73122 Inhibitors on Cell Viability, Neurite Outgrowth, and GAP-43 and CREB Protein Expression in SH-SY5Y Cells

**Figure 7:**
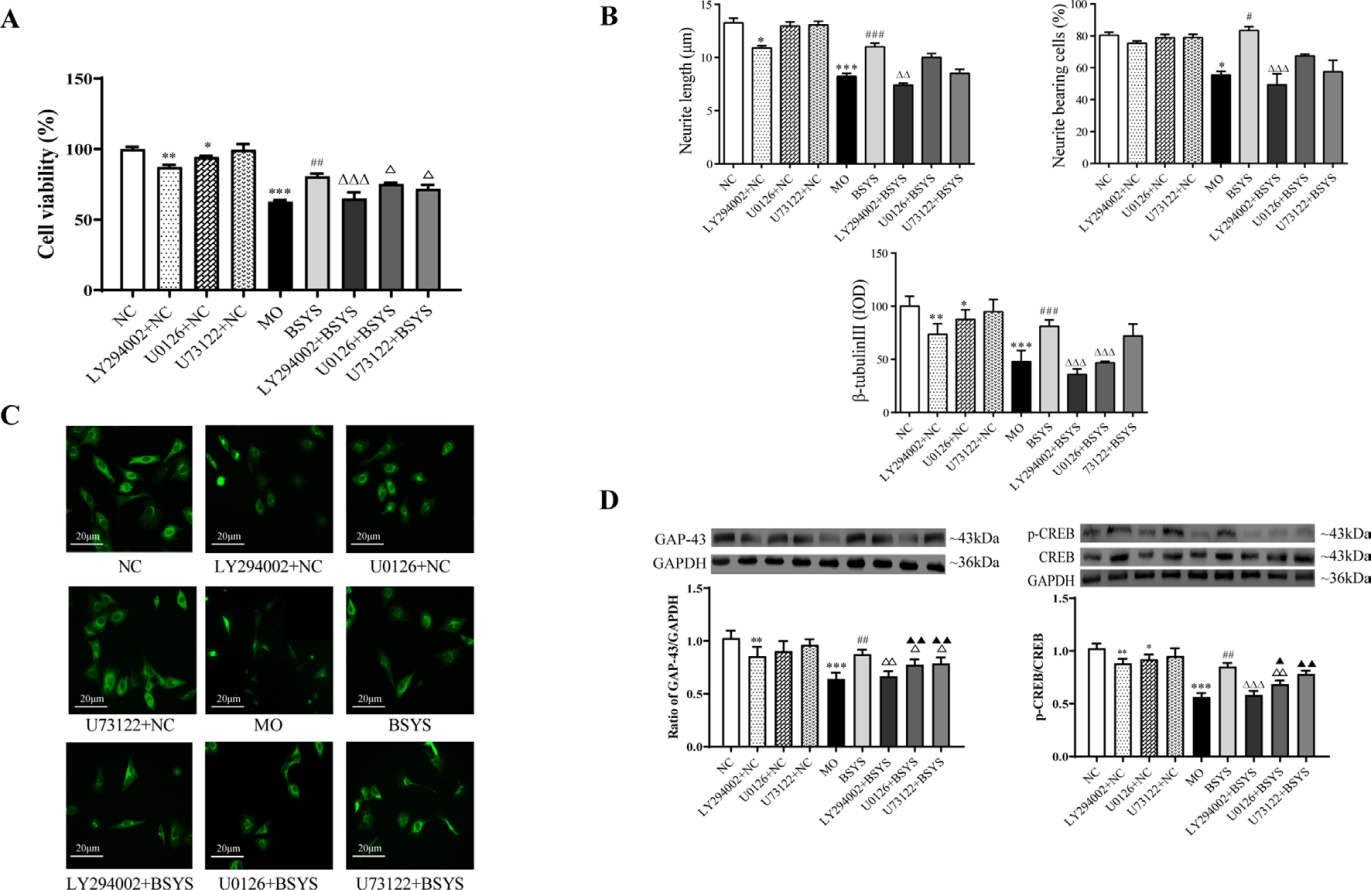
Effects of BSYS-containing serum and the inhibitors LY294002, U0126, and U73122 on cell viability, GAP-43, and CREB protein expression and neurite outgrowth in SH-SY5Y cells. SH-SY5Y cells were seeded in low-serum medium (0.25% FBS) for 24 h and then treated with medicine-free serum or serum containing medicines from BSYS for 24 h prior to exposure to the inhibitors LY294002 (60 µmol/L), U0126 (10 µmol/L), and U73122 (0.5 µmol/L) for 1 h. **(A)** Cell viability was determined by a CCK-8 assay, as described in the Materials and Methods, and expressed as a percentage relative to the control. **(B** and **C)** Neurite length, neurite-bearing cells, and the expression of β-tubulin-III were determined by an immunofluorescence (IF) assay (400×), as described in the Materials and Methods. **(D)** GAP-43 and p-CREB/CREB protein expression was detected by WB, as described in the Materials and Methods. Data represent the mean ± SE. *n* = 6. *P < 0.05, **P < 0.01, ***P < 0.001 vs. NC; ^#^ *P* < 0.05, ^##^ *P* < 0.01, ^###^ *P* < 0.001 vs. MO; ^△^ *P* < 0.05, ^△△^ *P* < 0.01, ^△△△^ *P* < 0.001 vs. BSYS; ^▲^ *P* < 0.05, ^▲▲^ *P* < 0.01, vs. LY294002 + BSYS.

**Figure 7A** shows that cell viability was significantly lower in the MO group than in the NC group (*P* < 0.001). Cell viability was significantly increased in the BSYS group compared to that in the MO group (*P* < 0.01). However, cell viability was significantly decreased in the LY294002 (PI3K inhibitor), U0126 (ERK inhibitor), and U73122 (PLCγ inhibitor) groups compared to that in the BSYS group (*P* < 0.001, *P* < 0.05), especially in the LY294002 group. As shown in **Figures 7B, C**, the neurite length, neurite bearing cells, and the IOD ratio of β-Tubulin-III in the MO group were significantly downregulated compared to those in the NC group (*P* < 0.001, *P* < 0.05, *P* < 0.001). However, the values of these three indexes were significantly upregulated in the BSYS group compared to those in the MO group (*P* < 0.001, *P* < 0.05, *P* < 0.001). The three indexes were also markedly suppressed by LY294002 treatment when compared to those in the BSYS group (*P* < 0.05, *P* < 0.001, *P* < 0.001). β-Tubulin-III expression in the U0126 group was markedly increased compared to that in the BSYS group (*P* < 0.01). As shown in **Figure 7D**, GAP-43 and p-CREB/CREB ratio in the MO group was significantly downregulated compared to that in the NC group (*P* < 0.001), and GAP-43 and p-CREB/CREB levels in the BSYS group were significantly upregulated compared to those in the MO group (*P* < 0.01). GAP-43 and p-CREB/CREB ratio in the LY294002 and U0126 groups were significantly downregulated compared to those in the BSYS group (*P* < 0.05, *P* < 0.01), and GAP-43 levels in the U73122 group were also significantly downregulated (*P* < 0.05). The inhibitory effect of LY294002 on GAP-43 and p-CREB/CREB ratio was stronger and more dramatic than that of U0126 or U73122 (*P* < 0.05, *P* < 0.01).

In addition, there were also significant changes in cell viability, neurite outgrowth, GAP-43, and p-CREB of three inhibitors added to the NC groups, particularly the LY294002 inhibitor.

### Effects of BSYS on Neurological Function Score of Mice With EAE

**Figure 8:**
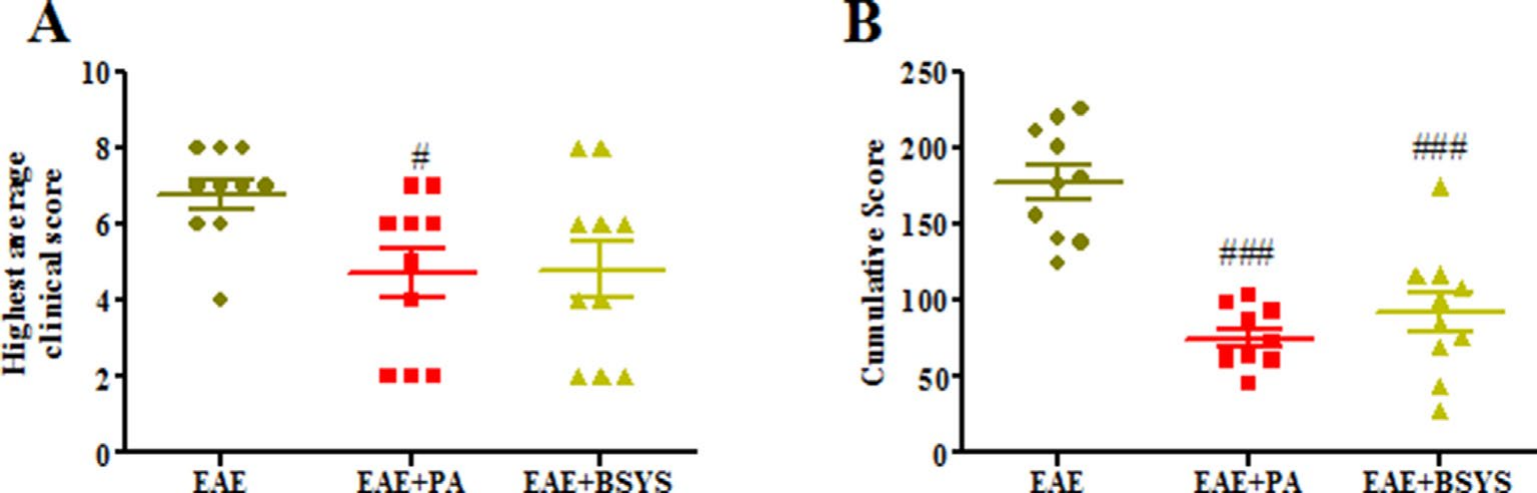
Effects of BSYS on neurological function score of mice with experimental autoimmune encephalomyelitis (EAE). Mice were scored daily until Day 40 PI. The scores were measured in EAE, EAE + PA, and EAE + BSYS. Data represent the mean ± SE (*n* = 10). **(A)** Highest clinical scores were measured on Days 16 PI. **(B)** Cumulative scores of different groups. ^#^ *P* < 0.05, ^###^ *P* < 0.001 vs. EAE.

Symptoms of EAE including flaccid tail, staggering gait, hind-limb paralysis, four-limb paralysis, and even death appeared sequentially in experimental mice from Day 6 PI. The highest clinical score was observed, and cumulative score were calculated. **Figure 8A** showed that the highest average score of PA-treated EAE mice were significantly decreased on Days 16 PI compared to the NC mice (*P* < 0.05). As shown in **Figure 8B**, the cumulative score was significantly downregulated with treatment of PA or BSYS compared to the NC mice (*P* < 0.001).

### Effects of BSYS on NF68, NF160, and NF200 Protein Expression in the Brain and Spinal Cord of Mice on Days 18 and 40 PI

**Figure 9:**
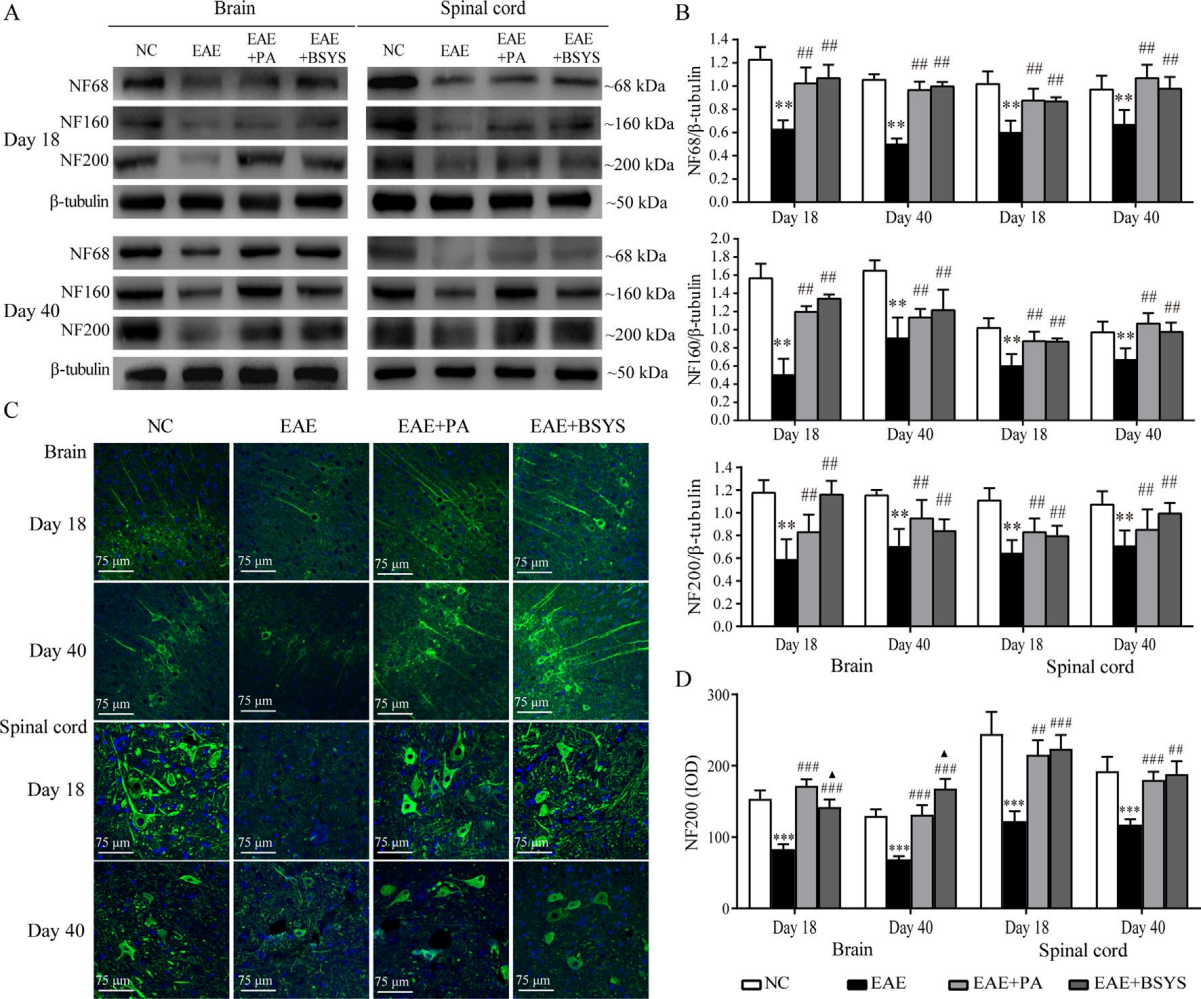
Effect of BSYS on NF68, NF160, and NF200 protein expression in the brain and spinal cord of mice on Days 18 and 40 PI. **(A** and **B)** NF68, NF160, and NF200 protein expression was detected by WB. **(C** and **D)** NF200 protein expression was also detected by IF, as described in the Materials and Methods. Data represent the mean ± SE, *n* = 5. ***P < 0.001 vs. NC; ^##^ *P* < 0.01, ^###^ *P* < 0.001 vs. EAE; ^▲^ *P* < 0.05 vs. EAE + PA.

To further confirm the mechanism through which BSYS functions, an animal experiment was carried out. NF68, NF160, and NF200 protein expression was detected with Western blot. As shown in **Figure 9A**, the Western blot analysis showed that NF68, NF160, and NF200 protein expressions were significantly decreased in the brain and spinal cord on Days 18 and 40 PI compared to that in the NC mice (*P* < 0.01). Treatment with PA or BSYS significantly increased NF68, NF160, and NF200 levels (*P* < 0.01). Moreover, NF200 protein expression was detected with IF. **Figure 9** shows that NF200 protein expression in the brain and spinal cord of EAE mice was dramatically decreased on Days 18 and 40 PI compared to that in the NC mice (*P* < 0.001), while treatment with PA or BSYS significantly increased NF200 levels (*P* < 0.01, *P* < 0.001). Compared to PA treatment, BSYS treatment also increased NF200 levels in the brain on Day 40 PI (*P* < 0.05).

### Effects of BSYS on BDNF and TrkB Protein and mRNA Expression Levels in the Brain and Spinal Cord of Mice on Days 18 and 40 PI

**Figure 10:**
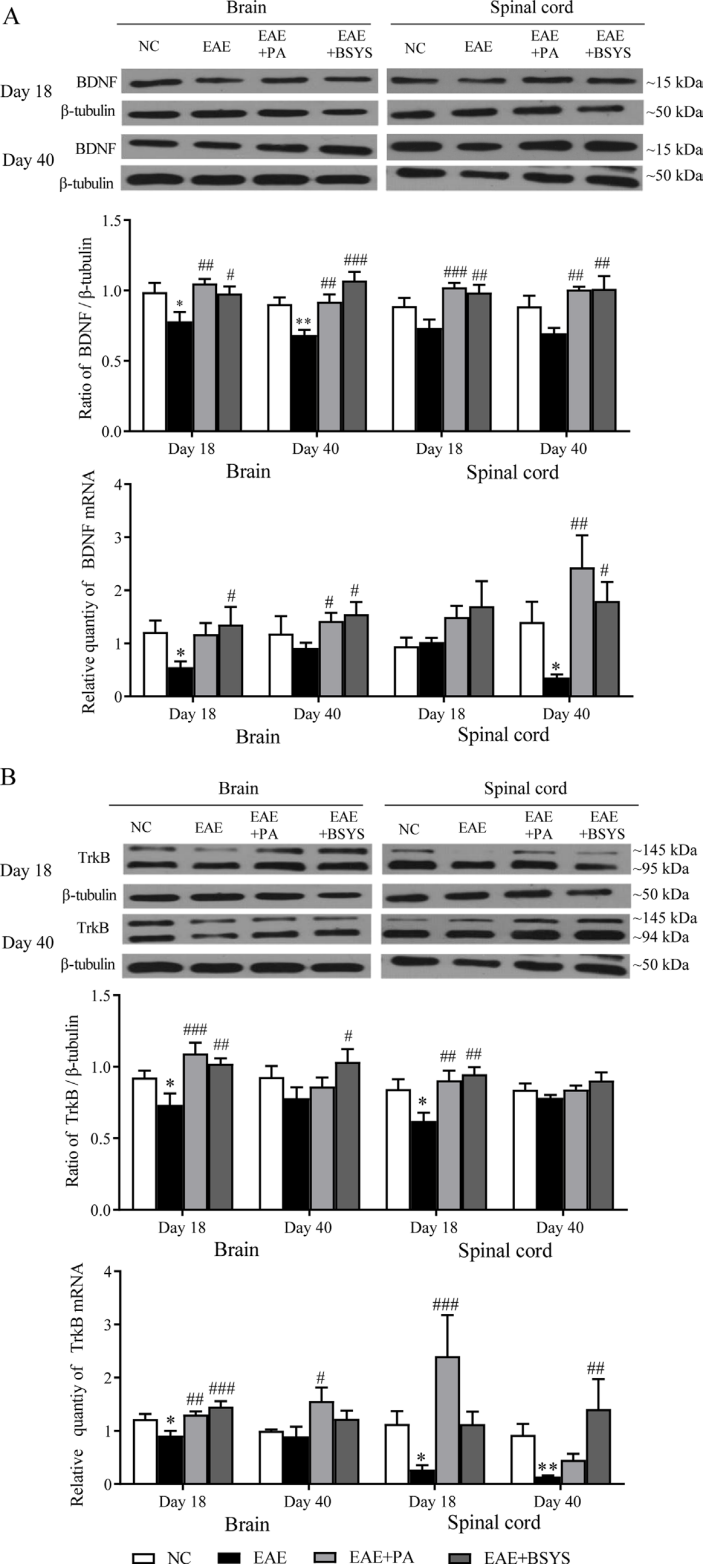
Effect of BSYS on BDNF and TrkB protein and mRNA expression in the brain and spinal cord of mice on Days 18 and 40 PI. BDNF **(A)** and TrkB **(B)** protein expression was detected by WB and quantitative reverse transcription polymerase chain reaction (qRT-PCR), as described in the Materials and Methods. Data are represented as the mean ± SE, *n* = 5 for each group. *P < 0.05, **P < 0.01 vs. NC; ^#^ *P* < 0.05, ^##^ *P* < 0.01, ^###^ *P* < 0.001 vs. EAE.

As shown in **Figure 10A**, the Western blot analysis showed that BDNF protein expression was significantly decreased in the brain on Days 18 and 40 PI compared to that in the NC mice (*P* < 0.05 and *P* < 0.01). In contrast, BDNF expression was markedly increased in the brain and spinal cord on Days 18 and 40 PI in PA- and BSYS-treated mice compared to that in EAE mice (*P* < 0.05, *P* < 0.01, *P* < 0.001). qRT-PCR analysis showed that BDNF mRNA expression was significantly decreased in the brain on Day 18 PI and in the spinal cord on Day 40 in the MO mice compared to that in the NC mice (*P* < 0.05, *P* < 0.05). In contrast, compared to that in the EAE mice, BDNF mRNA expression was significantly increased in the brain and spinal cord on Day 40 PI in the PA and BSYS mice (*P* < 0.05 and *P* < 0.01) and in the brain on Day 18 the PI in the BSYS mice (*P* < 0.05).

As shown in **Figure 10B**, the Western blot analysis showed that TrkB expression was markedly decreased in the brain and spinal cord on Day 18 PI in EAE mice compared to that in the NC mice (*P* < 0.05 and *P* < 0.01). In contrast, TrkB expression was markedly increased in the brain and spinal cord on Day 18 PI in the PA and BSYS mice compared to that in the EAE mice (*P* < 0.01, *P* < 0.001). In addition, BSYS also dramatically increased the TrkB levels in the brain on Day 40 PI (*P* < 0.05). qRT-PCR analysis showed that TrkB mRNA expression was markedly decreased in the brain on Day 18 PI and in the spinal cord on Days 18 and 40 PI compared to that in the NC mice (*P* < 0.05 and *P* < 0.01). In contrast, TrkB mRNA expression was significantly increased in the brain on Day 18 PI in the PA and BSYS mice, in the brain on Day 40 PI in the PA mice, and in the spinal cord on Days 18 PI and 40 PI in the BSYS mice compared to that in the EAE mice (*P* < 0.001, *P* < 0.01).

### Effects of BSYS on PI3K and Akt mRNA and Protein Expression in the Brain and Spinal Cord of Mice on Days 18 and 40 PI

**Figure 11:**
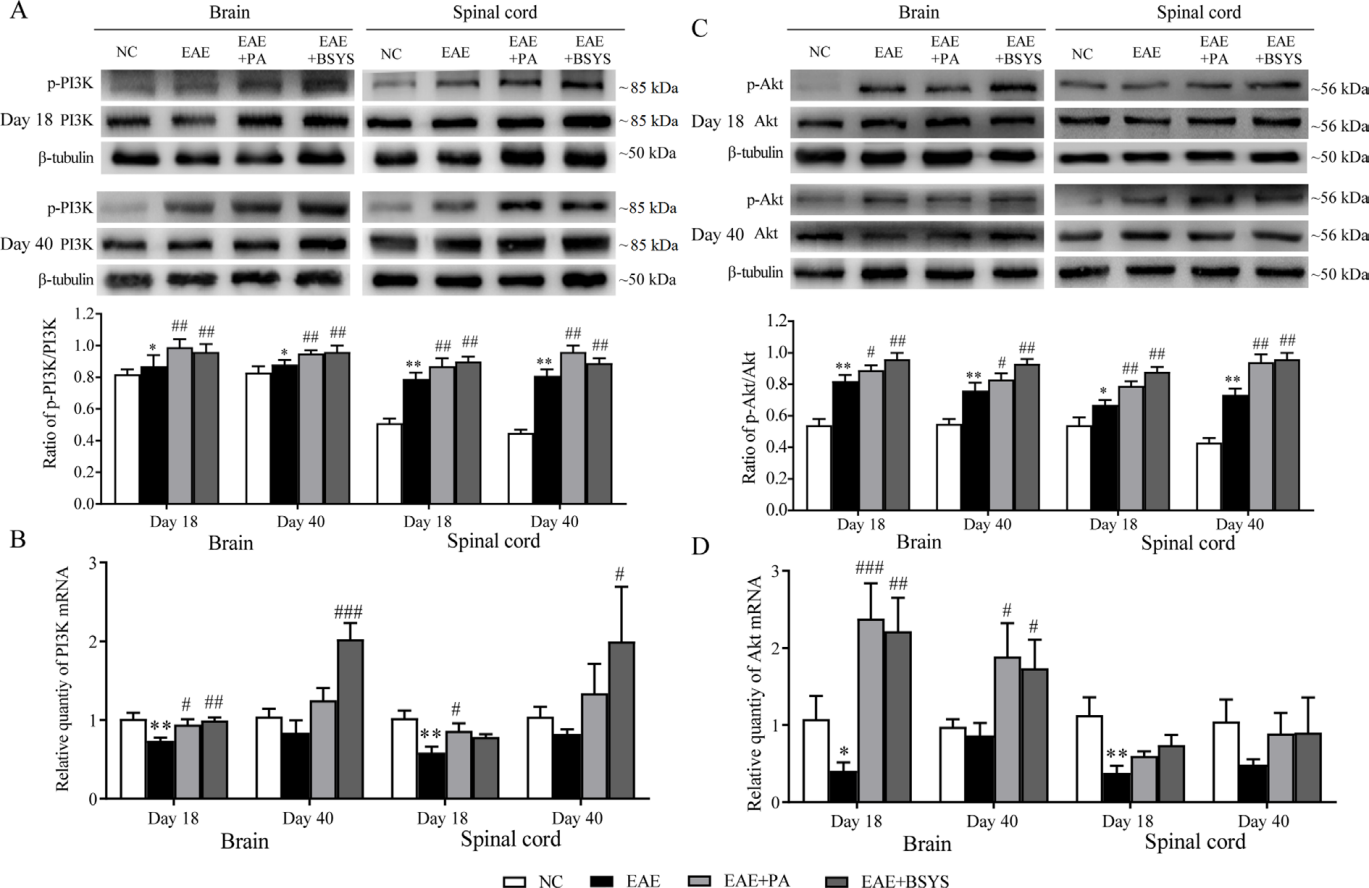
Effect of BSYS on PI3K and Akt protein and mRNA expression in the brain and spinal cord of mice on Days 18 and 40 PI. P-PI3K/PI3K **(A** and **B)** and p-Akt/Akt **(C** and **D)** protein expression and mRNA were detected by WB and qRT-PCR, as described in the Materials and Methods. Data are represented as the mean ± SE, *n* = 5 for each group. *P < 0.05, **P < 0.01 vs. NC; ^#^ *P* < 0.05, ^##^ *P* < 0.01, ^###^ *P* < 0.001 vs. EAE.

As shown in **Figure 11A**, the Western blot analysis showed that the p-PI3K/PI3K ratio was markedly increased in the brain and spinal cord on Day 18 and Day 40 PI in EAE mice compared to that in the NC mice (*P* < 0.05 and *P* < 0.01). The PA and BSYS treatments increased the p-PI3K/PI3K ratio in the brain and spinal cord of mice on Days 18 and 40 PI (*P* < 0.01). **Figure 11B** shows that PI3K mRNA expression was significantly decreased in the brain and spinal cord of the mice on Day 18 PI in EAE mice compared to that in the NC mice (*P* < 0.05 and *P* < 0.01, respectively). However, compared to that in the EAE mice, PI3K expression was markedly increased in the brain on Day 18 PI in the PA and BSYS mice and on Day 40 PI in the BSYS mice, and it was increased in the spinal cord on Day 18 PI in the PA mice and on Day 40 PI in the BSYS mice (*P* < 0.05, *P* < 0.01, and *P* < 0.001).

As shown in **Figure 11C**, the Western blot analysis showed that the p-Akt/Akt ratio was significantly increased in the brain and spinal cord on Day 18 and Day 40 PI in EAE mice compared to that in the NC mice (*P* < 0.05 and *P* < 0.01). The PA and BSYS treatments dramatically increased the p-Akt/Akt ratio in the brain and spinal cord of mice on Days 18 and 40 PI (*P* < 0.05 and *P* < 0.01). **Figure 11D** shows that Akt mRNA expression was significantly decreased in the brain and spinal cord on Day 18 PI in EAE mice compared to that in the NC mice (*P* < 0.05 and *P* < 0.01, respectively). The Akt mRNA was markedly increased in the brain on Days 18 and 40 PI in the PA- and BSYS-treated mice (*P* < 0.05, *P* < 0.01, and *P* < 0.001).

## Discussion

With long-term clinical studies, we found that BSYS treatment significantly lowered expanded disability status scale (EDSS) scores; alleviated or even eliminated the symptoms of limb weakness, pain, numbness, discharging, and banding sensation in MS patients; decreased the rate of recurrence and disability; reduced the side effects of PA; regulated immune function; and improved quality of life in MS patients. Magnetic resonance spectroscopy (MRS) results showed that BSYS promoted remyelination and the repair of nerve fiber bundles (Fang et al., 2013a; Zhou and Fan, 2015). In summary, it is more advantageous to treat MS patients with the integral regulating effect of this formula in the remission stage. BSYS also decreased neurological function scores, extended latency, reduced inflammatory cell infiltration and damage to axons and myelin, and promoted axon and myelin repair in EAE mice (Fang et al., 2017; Zhao et al., 2018). In vitro, BSYS-containing serum significantly increased cell viability and neurite outgrowth in SH-SY5Y cells cultured in low-serum medium (Liu et al., 2014; Zheng et al., 2015). Based on the findings above, we hypothesized that BSYS has neuroprotective effects in patients with MS and its experimental models. However, the molecular mechanisms through which BSYS functions remain unclear.

To clarify the mechanism of this Chinese medicine formula in the current study, BSYS was decomposed into two parts, BS and HTHX. The effects of BSYS, BS, and HTHX on axonal regeneration and their mechanisms of action on neurotrophic factors and related signaling pathways were explored.

This study found that GAP-43 is a protein specific to the nerve cell membrane, and it is closely associated with neurodevelopment, central synapse remodeling, and axonal regeneration. GAP-43 has been recognized as an internal decisive factor for nerve growth, recovery, and regeneration (Kato et al., 2013). CREB is an important nuclear transcription factor in eukaryotes, and p-CREB plays a critical role in neural regeneration and synapse formation (Liu et al., 2017; Jana et al., 2018).

In *in vitro* studies, we observed that BSYS promoted survival and axonal outgrowth and markedly increased the expression of the GAP-43 and CREB proteins after 24 h of treatment in SH-SY5Y cells. In addition, BS and HTHX both individually increased GAP-43 expression. These results imply that BSYS and its decomposed forms, BS and HTHX, promote axonal regeneration, and the effect of BSYS was superior to that of BS or HTHX.

Many factors are known to be involved in the remyelination and axonal regeneration process. A lack of neurotrophic factors negatively affects CNS regeneration. To study further the mechanism by which BSYS, BS, and HTHX promote axonal regeneration, BDNF, TrkB, and their related signaling pathways, including PI3K, ERK, and PLCγ, were investigated. BDNF is one of the most important neurotrophic factors, and it influences synaptic plasticity and participates in neuronal growth, development, and apoptosis prevention (Zhang et al., 2018). Together with its TrkB receptors in the axon terminals, BDNF maintains and promotes neuronal differentiation, growth, and regeneration, and it also provides nutrition for injured neurons and helps recover injured sensory and motor neurons (Li et al., 2015). TrkB activation initiates three major intracellular signaling cascades—PI3K/Akt, MEK/ERK, and PLCγ (Reichardt, 2006; Agosto-Marlin and Mitchell, 2017). The activated PI3K/Akt pathway not only promotes cell survival and inhibits apoptosis but also plays a critical role in axons (Vallée et al., 2018). The MEK/ERK pathway plays an important role in cell proliferation and differentiation (Gokce et al., 2009; Jiang et al., 2011). Ca^2+^ accumulation has an important effect on the physiology and structure of the cytoskeleton. The PLCγ pathway increases Ca^2+^ release from the intracellular pool. Thus, these three signaling pathways play important roles in axonal regeneration in MS. Published research has reported that BDNF and its receptors are increased around lesions in MS, indicating that BDNF participates in focal repair (Stadelmann et al., 2002). Another study showed that BDNF and TrkB are downregulated during MS/EAE (Vacaras et al., 2014; Rajendran et al., 2018). Our previous studies found that the expression of BDNF in the brain and spinal cord is reduced in mice with EAE (Zheng et al., 2013). In this study, BSYS treatment increased BDNF and TrkB protein expression in SY5Y cells; however, there were no significant effects of BS and HTHX on BDNF and TrkB levels. Our study also found that treatment with BSYS significantly increased PI3K protein expression, and BS or HTHX treatment significantly increased ERK protein expression. However, the BSYS, BS, and HTHX treatments had no effects on PLCγ protein levels. According to the above results, we speculated that the mechanism by which BSYS promotes axonal regeneration is related to the PI3K/Akt pathway and that BS and HTHX act through the ERK pathway. The overall regulatory effects of BSYS were better than those of BS and HTHX.

To further verify the targets of the formula, the possible involvement of the PI3K, ERK, and PLCγ signaling pathways was investigated using their molecular inhibitors. SH-SY5Y cells were incubated with low-serum medium and subsequently treated with LY294002, U0126, and U73122, which are inhibitors of the PI3K, ERK, and PLCγ pathways, respectively, before treatment with BSYS-containing serum. Cell viability was markedly reduced by approximately 25%, 13%, and 20%, respectively, after these treatments. LY294002 almost completely blocked BSYS-induced neurite outgrowth and expression of GAP-43 and CREB in SH-SY5Y cells. U0126 and U73122 only partially inhibited the effects of BSYS. This result implies that the PI3K pathway may contribute to the ability of BSYS to promote axonal regeneration, and the ERK pathway may also participate in this neurogenic activity.

Furthermore, we carried out *in vivo* experiments to observe the effects of BSYS on axonal regeneration in EAE mice. NFs are one of the major components of the neuronal cytoskeleton, and they are essential for the radial growth and structural stability of myelinated axons. Mature axons maintain a high density of NFs of three different molecular weights: NF68, NF160, and NF200 kDa (Yuan et al., 2017). An increase in NF200 levels indicates that more nerve fibers are present (Zhao et al., 2018). Our study found that BSYS markedly increased the levels of NF68, NF160, and NF200 in the brain and spinal cord of mice, indicating that BSYS increased nerve fibers in axons. This study also showed that BSYS increased the mRNA and protein levels of BDNF/TrkB and PI3K/Akt in the brain and spinal cord at different time points (Day 18 or 40 PI). The above results demonstrated that BSYS-induced recovery of neurological function occurred through increased expression of BDNF and TrkB through the PI3K/Akt pathway to regulate nerve repair. BSYS was more effective than PA, especially in the brain on Day 18 PI and in the spinal cord on Day 40 PI.

Many herbal medicine components of the BSYS formula have extensive neuroprotective and immunoregulatory effects. For example, echinacoside (*Radix Rehmanniae*) inhibits inflammatory processes and activates the Akt/GSK-3β pathway (Benveniste et al., 2018), and 2,3,5,4′-tetrahydroxyl diphenylethylene-2-O-glucoside (*Radix Rehmanniae*) enhances protection in an ischemia/reperfusion model of HUVECs (Liu et al., 2010). Recent research has reported that *Radix Rehmanniae* ameliorates EAE by suppressing macrophage-derived nitrative damage (Li et al., 2018). Forsythoside B and forsythoside D (*Fructus Forsythiae*) inhibit PC12 cell damage induced by rotenone and increase cell viability (Zhang et al., 2015a). Additionally, the remarkable anti-inflammatory and antioxidant capacities of *Forsythiae Fructus* contribute to its neuroprotective activities (Wang et al., 2018). Thus, these components represent the material basis for the observed neuroprotective functions.

### Conclusions

In this study, the possible involvement of BSYS and its decomposed BS and HTHX formulas in the PI3K, ERK, and PLCγ signaling pathways was investigated in SH-SY5Y cells. The results showed that the regulation of BSYS was related to the PI3K/Akt pathway. Therefore, we focused on the PI3K/Akt pathway to further verify the mechanism of the BSYS formula in EAE mice. The above *in vitro* and *in vivo* experimental results revealed that BSYS had significantly promoted axonal regeneration, and its mechanism was related to the regulation of the BDNF/TrkB and PI3K/Akt pathways. BS and HTHX also promoted nerve regeneration, and the ERK pathway was involved in their activity. These findings provide strong experimental evidence supporting this therapeutic method combining herbal medicines that tonify the kidney, resolve phlegm, and activate stasis for the treatment of MS.

## Ethics Statement

This study was carried out in accordance with the recommendations of the Guide for Laboratory Animal Care. The protocol was approved by the Ethics Committee of Capital Medical University (No. 2011-X-001).

## Author Contributions

QZ, LL, TY, HL, HZ, HS, JJ, and YS participated in the experiments. QZ, LL, and HL analyzed the data and wrote the paper. LW and YF secured the funding, designed the experiments, and revised the paper. HZ provided technical guidance. FQ, KL, JL, and NZ participated in experimental preparation and statistical analysis. All authors read and approved the final manuscript.

## Funding

This work was supported by the National Natural Science Foundation of China (No. 81873252, No. 81573898, and No. 81473640), the Beijing Natural Science Foundation (No. 7182020), and the Program of Changcheng Scholars for the Importation and Development of High-Caliber Talents Project of Beijing Municipal Institutions (CIT&TCD20140329).

## Conflict of Interest Statement

The authors declare that the research was conducted in the absence of any commercial or financial relationships that could be construed as a potential conflict of interest.
